# Physical Activity Characteristics across GOLD Quadrants Depend on the Questionnaire Used

**DOI:** 10.1371/journal.pone.0151255

**Published:** 2016-03-14

**Authors:** Heleen Demeyer, Elena Gimeno-Santos, Roberto A. Rabinovich, Miek Hornikx, Zafeiris Louvaris, Willem I. de Boer, Niklas Karlsson, Corina de Jong, Thys Van der Molen, Ioannis Vogiatzis, Wim Janssens, Judith Garcia-Aymerich, Thierry Troosters, Michael I. Polkey, PROactive consortium

**Affiliations:** 1 KU Leuven, Department of Rehabilitation Sciences, B-3000, Leuven, Belgium; 2 University Hospitals Leuven, Department of Respiratory Diseases, B-3000, Leuven, Belgium; 3 Centre for Research in Environmental Epidemiology (CREAL), Barcelona, Spain; 4 CIBER Epidemiologia y Salud Publica (CIBERESP), Barcelona, Spain; 5 Universitat Pompeu Fabra (UPF), Barcelona, Spain; 6 ELEGI/Colt laboratory, UoE/MRC Centre for Inflammation Research, The University of Edinburgh, Edinburgh, Scotland; 7 Dept of Critical Care Medicine, Pulmonary Rehabilitation Centre, Evangelismos Hospital, M. Simou and G.P. Livanos Laboratories, National and Kapodistrian University of Athens, Thorax Foundation, Athens, Greece; 8 Department of Pulmonology, Leiden University Medical Center, Leiden, Netherlands; 9 Astra Zeneca, Mölndal, Sweden; 10 Department of General Practice, University Medical Center Groningen, Groningen, Netherlands; 11 Department of Primary Care, University of Groningen, University Medical Centre Groningen, Groningen, Netherlands; 12 NIHR Respiratory Biomedical Research Unit of the Royal Brompton and Harefield NHS foundation Trust and Imperial College London, London, United Kingdom; University of Pittsburgh, UNITED STATES

## Abstract

**Background:**

The GOLD multidimensional classification of COPD severity combines the exacerbation risk with the symptom experience, for which 3 different questionnaires are permitted. This study investigated differences in physical activity (PA) in the different GOLD quadrants and patient’s distribution in relation to the questionnaire used.

**Methods:**

136 COPD patients (58±21% FEV_1_ predicted, 34F/102M) completed COPD assessment test (CAT), clinical COPD questionnaire (CCQ) and modified Medical Research Council (mMRC) questionnaire. Exacerbation history, spirometry and 6MWD were collected. PA was objectively measured for 2 periods of 1 week, 6 months apart, in 5 European centres; to minimise seasonal and clinical variation the average of these two periods was used for analysis.

**Results:**

GOLD quadrants C+D had reduced PA compared with A+B (3824 [2976] vs. 5508 [4671] steps.d-1, p<0.0001). The choice of questionnaire yielded different patient distributions (agreement mMRC-CAT κ = 0.57; CCQ-mMRC κ = 0.71; CCQ-CAT κ = 0.72) with different clinical characteristics. PA was notably lower in patients with an mMRC score ≥2 (3430 [2537] vs. 5443 [3776] steps.d^-1^, p <0.001) in both the low and high risk quadrants.

**Conclusions:**

Using different questionnaires changes the patient distribution and results in different clinical characteristics. Therefore, standardization of the questionnaire used for classification is critical to allow comparison of different studies using this as an entry criterion.

**Clinical Trial Registration:**

ClinicalTrials.gov NCT01388218

## Introduction

Chronic Obstructive Pulmonary Disease (COPD) is the most common lung disease in the world. In 2011, the Global Initiative for Chronic Obstructive Lung Disease (GOLD) strategy proposed new recommendations for the assessment and management of patients with COPD, using a multidimensional approach to classify patients into four quadrants. This classification has the advantage of combining the risk of exacerbations, based on both lung function and exacerbation history, with the symptoms experienced by patients [1]. It was hypothesised that this quadrant classification would provide a better reflection of the complexity of COPD compared to the uni-dimensional analysis of airflow limitation previously used for staging the disease.

The stated goals of COPD assessment are to determine the severity of the disease, its impact on the patient’s health status and the risk of future events, to ultimately guide therapy [2]. This strategy provoked a release of new publications, the so called “GOLD rush” [3], mainly investigating the classifications ability to predict future events, such as exacerbations, hospital admissions and death. However two large combined datasets have shown that at least for the prediction of death it offers no advantage over the preceding I-IV stage classification [4,5].

Physical activity (PA) has emerged as a strong–if not the strongest- predictor of both mortality and risk of hospitalization due to a COPD exacerbation [6]. PA is of intuitive relevance to quality of life and is decreased in patients with COPD compared to healthy people [7]. Using the prior I-IV GOLD stage system it was possible to show a relationship, albeit not a strong relationship, between PA and FEV_1_ [8,9]. A lower level of PA has previously been described even in patients with GOLD stage I [10] or in newly diagnosed patients with COPD [11]. Increasing PA has become an additional non-pharmacological component of the recommended treatment of patients with COPD, in addition to pulmonary rehabilitation.

In the 2013 GOLD recommendations, the COPD Assessment Test (CAT), the modified Medical Research Council (mMRC) questionnaire or the Clinical COPD Questionnaire (CCQ) can all be used for assessing symptoms. Previous research concluded that the quadrant distribution of patients classified by the mMRC, CAT or CCQ score is not identical [12,13], but it is not yet known whether the quadrant construct can differentiate patients based on their PA.

Therefore the aim of the present study is to investigate whether the new GOLD classification captures differences in PA. For that purpose we posed two specific questions; first, what are the patients’ PA characteristics according to the different classifications and second, what is the agreement between the different classifications?

## Methods

### Study population and design

As part of the PROactive project (www.proactivecopd.com, NCT01388218) 236 patients with COPD [2] were followed during 1 year with repeated assessments in between. The present non-interventional cross-sectional study is based on patients who completed the whole trial and uses data captured during the interim and final visit, respectively 6 and 12 months after the screening visit, that allowed prospective assessment of exacerbations. All patients signed a written informed consent before starting any data collection. This study was approved by the PROactive ethics and patient advisory boards and approved by the ethics committee at each centre (Commissie medische ethiek van de universitaire ziekenhuizen KU Leuven; Medische ethische toetsingscommissie universitair medisch centrum Groningen; Lothian ethics committee; South east Scotland research ethics committee; Scientific council of the general hospital for chest diseases, Sotiria).

Patients were recruited from five European clinical centres [Leuven (Belgium), London and Edinburgh (UK), Athens (Greece) and Groningen (The Netherlands)], participating in the PROactive project. These centres were chosen to ensure geographical and cultural representation of a wide range of disease severity by comprising primary care, tertiary care and rehabilitation centres. Patients had a smoking history of at least 10 pack years and were not suffering from a respiratory disease other than COPD. Patients with orthopaedic or neurological complaints which would limit PA and patients with cognitive impairment were excluded. More detailed information has been described in detail elsewhere [14].

### Clinical measurements

All patients underwent spirometry according to ERS-ATS standards [15] to confirm the diagnosis of COPD (screening visit) and to be used as part of the GOLD classification (final visit). A 6-minute walk test (6MWD) was performed during the final visit in a 30m corridor, using the best of 2 tests [16]. Results were expressed as a percentage of predicted normal values [17,18].

### Physical activity

PA was measured with the Dynaport Movemonitor (Mc Roberts BV, The Hague, The Netherlands) for two periods: 1 week preceding the interim visit and 1 week preceding the final visit. The mean of both measurements (6 months apart) was used as an outcome measurement, with the aim of lowering the influence of individual changes due to the weather and patient condition during 1 single measurement. Patients were asked to wear the monitor during waking hours. The tri-axial accelerometer, worn at the height of the second lumbar vertebra has been thoroughly validated in patients with COPD [19,20].

Valid days were defined as days with a wearing time of at least 8 hours, weekends were excluded from the analysis, resulting in two periods of (maximum) five weekdays. Patients with at least 2 valid days at both time points were included in the analysis. The measurement of 2 weekdays will provide the desirable intraclass correlation coefficient (≥0.80) for cross-sectional analyses [21]. To account for potential misclassification in relation to decisions about PA, all analyses were repeated using all patients with an overall (either of both measurements) minimum of 2 days (week or weekend) of PA data (S1 File). The total amount of steps per day and the proportion of patients showing inactivity (mean daily step value of <4580 steps [22]) were chosen as outcome measurements.

### Questionnaires

The GOLD strategy recommends the use of either CAT, mMRC or the CCQ questionnaire for the symptomatic assessment. The CAT measures the impact of COPD on a person’s health status [23], a score ≥10 is defined as having “high” symptoms in the GOLD strategy. The mMRC assesses the disability due to breathlessness [24] where an mMRC grade ≥ 2 points indicates having “high” symptoms in the current recommendation. The CCQ is developed to measure symptom and functional status [25]. The cut-off for CCQ has to be finally determined in the GOLD strategy but will be in the range 1–1.5. The present study considered a cut-off of ≥1.5 [26].

### Exacerbation history

Acute exacerbations were defined as ‘events in the natural course of the disease, characterized by a change in the patients’ baseline dyspnea, cough and/or sputum, which is beyond normal day-to-day variations, acute in onset and leading to a change in medication’[1]. Having two or more exacerbations in the preceding year, or having at least one hospitalization for a COPD exacerbation in the preceding year was defined as ‘at high risk’.

### Definition of GOLD quadrants

**Fig 1:**
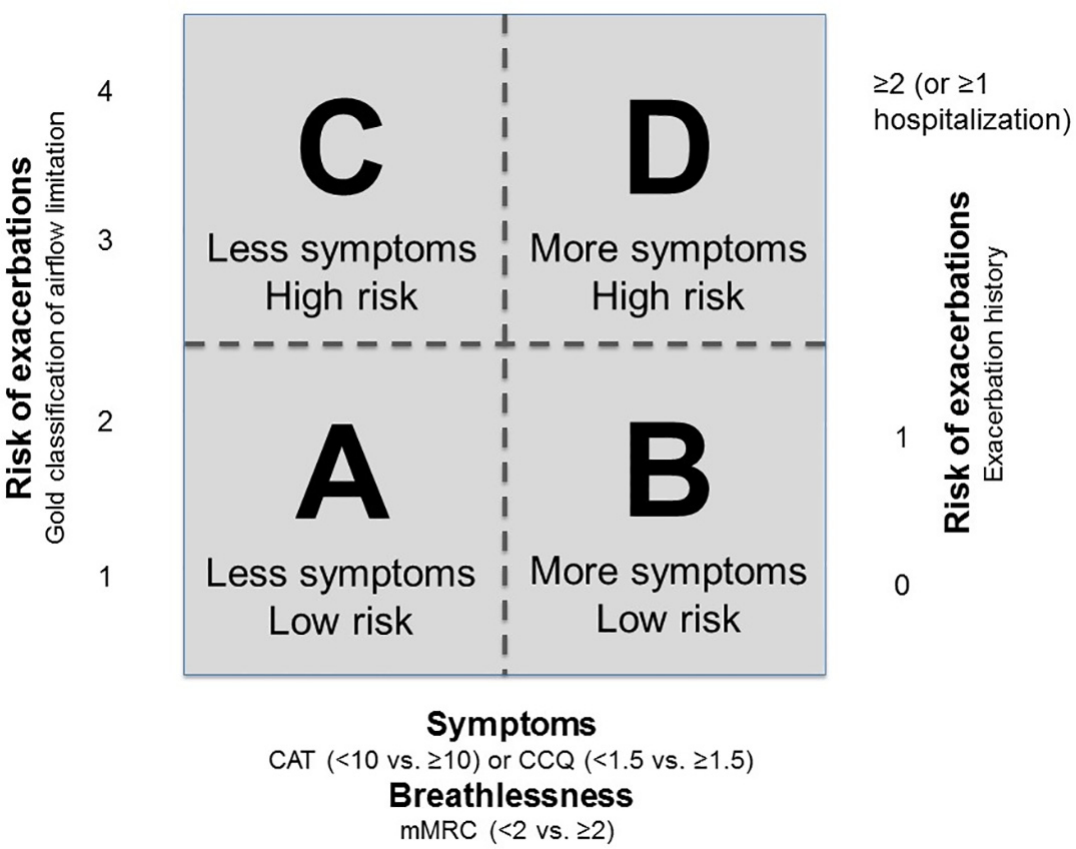
The new GOLD assessment. The classification combines the symptomatic assessment with the patients’ spirometric classification and/or risk of exacerbations, resulting in 4 quadrants. To classify patients first symptoms should be assessed with the mMRC, CAT or CCQ scale and investigator should determine if the patient belongs to the left (less symptoms) or right (more symptoms) side of the box. Next the risk of exacerbations should be assessed to determine if the patient belongs to the lower part of the box (low risk) or the upper part of the box (higher risk). When assessing symptoms the mMRC, CAT or the CCQ scale can be used; when assessing risk the highest risk according to GOLD grade or exacerbation history should be chosen.

The combined GOLD classification categorizes patients in 4 quadrants (A-D) taking into account lung function, exacerbation history and symptoms [1]. The classification is in detail described in Fig 1.

### Statistics

Sample size was defined according to the main objective of the study [14]. We calculated whether the available sample size provided enough statistical power to answer the research question(s), concluding this sample sufficient (S1 File). Analyses were performed with the SAS statistical package 9.3 (SAS institute, Cary, NC, USA) and GraphPad Prism 6.01 (GraphPad Software, Inc, CA, USA). The normality of distribution was analyzed with the Shapiro-Wilk test and visual inspection of the frequency distribution. Data are presented as mean±SD for normally or median [interquartile range] for not normally distributed variables. Statistical significance was set at p<0.05. The different research questions were answered based on the following analyses:

1. What are the patients’ characteristics according to the different classifications?Differences in patient characteristics across the former stages and GOLD quadrants were analyzed using a one way ANOVA (proc GLM) for continuous variables (chi square tests for categorical variables) followed by a post hoc analysis (Tukey), if significant. Differences in daily step count were analyzed using a Kruskal Walis test with post hoc pairwise comparison (Mann-Whitney U), applying a Dunn’s correction for multiple testing. Because we hypothesized a low number of patients in quadrant C, we pooled patients in quadrant A+C and B+D to investigate the impact of symptoms on PA (Mann-Whitney U). Similarly, the difference in physical activity between patients in the upper quadrants (C+D) and stages (III+IV) were compared to those of patients in the lower quadrants (A+B) and stages (I+II).
2. What is the agreement between the different classifications using different definitions? Agreement between the classifications (quadrants A to D), based on either CAT, mMRC or CCQ questionnaire results, was investigated using a (pairwise) concordance analysis. A simple kappa–coefficient (κ) was calculated (κ>0.40 indicates moderate agreement; κ>0.60 indicates substantial agreement; κ>0.80 indicates almost perfect agreement[27]).

## Results

### General patient characteristics

A total of 191 patients with COPD completed the whole trial [14] and were considered for the present study (S1 Fig). Twenty two patients had missing data of the GOLD stratifiers (mMRC, CAT, CCQ or exacerbation history) and 33 additional patients had invalid PA data, resulting in a total sample size of 136 patients (102 men, 34 women, 58±21% FEV_1_ predicted, 68±8 year) for the present analysis, see Table 1. Patients included in the analysis did not differ from those excluded based on insufficient valid PA data (Table A in S1 File). Patients wore the accelerometer for a total of 8.7±1.3 valid (week)days, with a mean wearing time of 881±107 minutes. In total 75 patients were allocated to C/D, of these 25 patients were categorized at high risk only based on their lung function, 25 patients solely based on their exacerbation history (≥2 exacerbations /≥1admission) and 25 based on both severe airflow limitation and the exacerbation history.

**Table 1:** Patient characteristics.

Variable	Patient characteristics (n = 136)
Female/male*	34 (25) / 102 (75)
Age (y)	68 ± 8
BMI (kg.m^-2^)	27 ± 5
FEV_1_ (%pred)	58 ± 21
6MWD (m)	444 ± 129
CAT score	14 ± 8
CCQ score	1.8 ± 1.0
mMRC (0/1/2/3/4)*	19 (14) / 53 (39) / 36 (26) / 27 (20) / 1 (1)
Active smokers*	18 (13)
COPD exacerbations (n.y^-1^)^&^	1 (0–12)
Hospitalizations due to COPD exacerbation (n.y^-1^) ^&^	0 (0–4)

Data are presented as mean ± SD

*data presented as n (%)

^&^data presented as median (min-max)

BMI missing in 2 patients, 6MWD missing in 6 patients

### What are the patients’ characteristics according to the different classifications?

The multidimensional classification as well as the spirometric GOLD stages could discriminate for all GOLD stratifiers (FEV_1_%_pred_, mMRC, CAT, CCQ, exacerbation history), see Table 2 and Table B in S1 File. The different quadrants nor GOLD stages did differ in age, gender, BMI, proportion of active smokers and wearing time of the activity monitor.

**Table 2:** Patient characteristics across the different GOLD quadrants using mMRC, CAT or CCQ score to define symptoms experience.

**Variable**	**Combined assessment (mMRC)**				
	A (n = 46)	B (n = 15)	C (n = 26)	D (n = 49)	p- value
FEV_1_ (%pred)	74±15	66±17	55±18 ^A^	42±18 ^A^^B^	**<0.01**
mMRC (score)	0.7±0.5	2.4±0.5^A^	0.9±0.3 ^B^	2.5±0.5 ^A^^C^	**<0.01**
CAT (score)	10±5	17±8^A^	11±6 ^B^	19±7 ^A^^C^	**<0.01**
CCQ (score)	1.1±0.7	2.1±0.9 ^A^	1.4±0.8 ^B^	2.5±0.8 ^A^^C^	**<0.01**
Exacerbations (n.y^-1^)^&^	0[0]	0[1]	2[1] ^A^^B^	3[3]^A^^B^	**<0.01**
Hospitalizations (n.y^-1^)^&^	0	0	0[0] ^A^^B^	0[0]^A^^B^	**<0.01**
6MWD (m)	523±98	349±96 ^A^	498±100^B^	367±115 ^A^^C^	**<0.01**
6MWD (%pred)	83±15	55±12 ^A^	79±14^B^	60±18 ^A^^C^	**<0.01**
Inactive patients (%)*	16 (35)	11(73) ^A^	14 (54)	39 (80)^A^^C^	**<0.01**
**Variable**	**Combined assessment (CAT)**				
	A (n = 27)	B (n = 34)	C (n = 16)	D (n = 59)	p- value
FEV_1_ (%pred)	75±17	70±15	50±20 ^A^^B^	46±18 ^A^^B^	**<0.01**
mMRC (score)	0.7±0.7	1.4±0.9 ^A^	1.4±0.8	2.1±0.9 ^A^^B^^C^	**<0.01**
CAT (score)	6±2	16±5 ^A^	6±3 ^B^	19±7 ^A^^B^^C^	**<0.01**
CCQ (score)	0.8±0.6	1.8±0.7 ^A^	1.1±0.6 ^B^	2.4±0.9 ^A^^B^^C^	**<0.01**
Exacerbations (n.y^-1^)^&^	0[0]	0[1]	1[2]	2[3]^A^^B^	**<0.01**
Hospitalizations (n.y^-1^)^&^	0	0	0[0]	0[0]^A^^B^	**<0.01**
6MWD (m)	519±111	452±126	449±149	404±118^A^	**<0.01**
6MWD (%pred)	83±18	71±7	72±20	65±18^A^	**<0.01**
Inactive patients (%)*	9 (33)	18 (53)	12 (75) ^A^	41 (69) ^A^	**<0.01**
**Variable**	**Combined assessment (CCQ)**				
	A (n = 40)	B (n = 21)	C (n = 16)	D (n = 59)	p- value
FEV_1_ (%pred)	75±15	67±15	54±21^A^	44±17^A^^B^	**<0.001**
mMRC (score)	0.8±0.7	1.6±1.0^A^	1.0±0.7	2.2±0.8 ^A^^B^^C^	**<0.001**
CAT (score)	9±4	16±7 ^A^	8±4^B^	19±7 ^A^^C^	**<0.001**
CCQ (score)	0.8±0.3	2.4±0.5 ^A^	0.9±0.4 ^B^	2.5±0.8 ^A^^C^	**<0.001**
Exacerbations (n.y^-1^)^&^	0[0.5]	0[1]	1[2]	2[3]^A^^B^	**<0.001**
Hospitalizations (n.y^-1^)^&^	0[0]	0[0]	0[0]	0[0]^A^^B^	**<0.001**
6MWD (m)	513±106	416±134 ^A^	485±137	394±116 ^A^^C^	**<0.001**
6MWD (%pred)	81±16	65±18^A^	74±17	64±19^A^^C^	**0.001**
Inactive patients (%)*	15(38)	12(57)	10(63)	43(73)^A^	**0.006**

Data are presented as mean ± SD

* = data presented as n (%)

^&^data presented as median [IQR]

p-value results from a one-way ANOVA, chi square test(*) or Kruskal wallis test (^&)^; Post hoc analyses, adjusted for multiple testing

^A^different from A (B, C or D)

^B^different from B (C or D)

^C^different from C (D)

**Fig 2:**
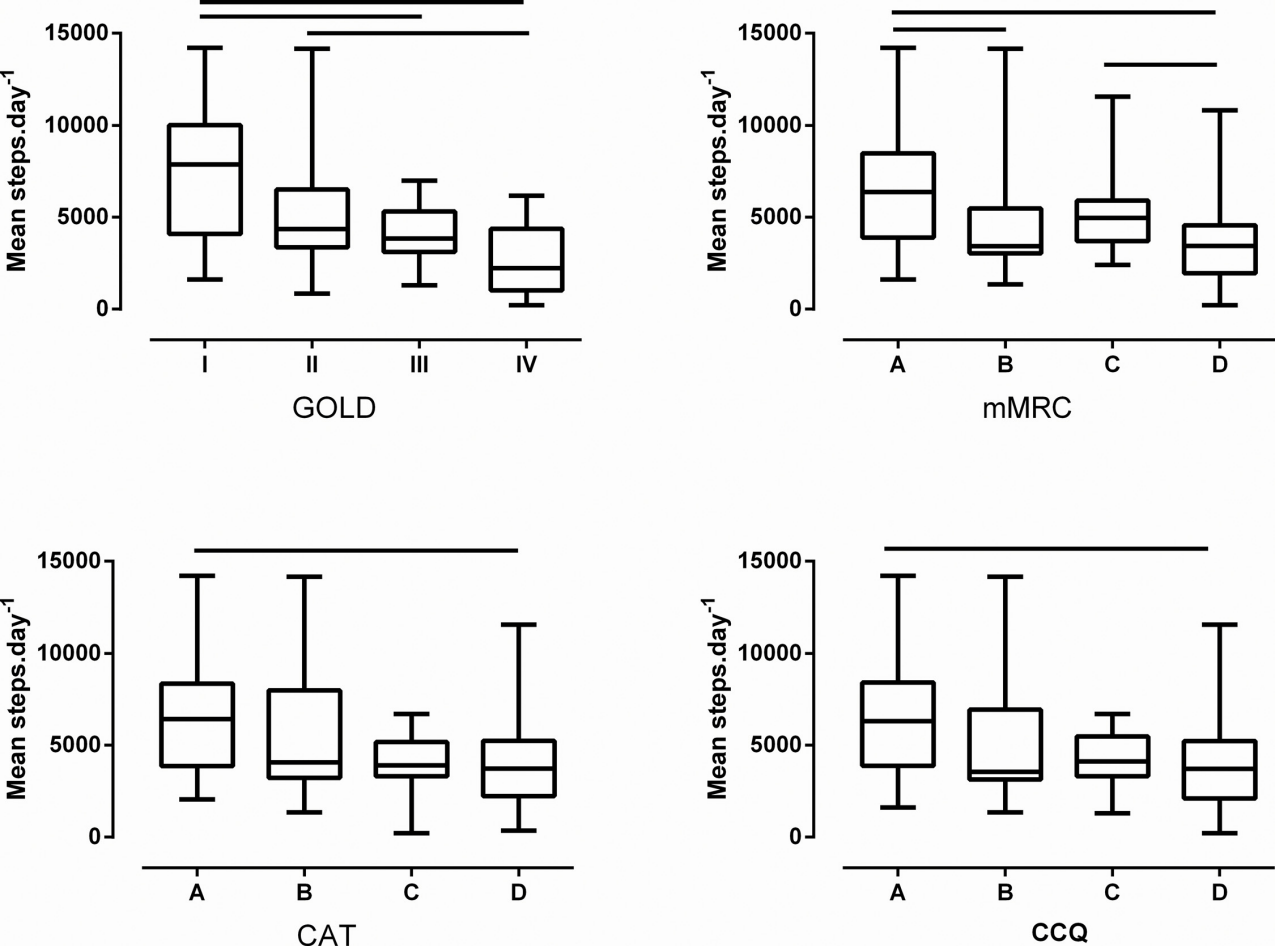
Physical activity across different GOLD classifications. GOLD = Spirometric GOLD classification; mMRC = combined assessment using mMRC; CAT = combined assessment using CAT; CCQ = combined assessment using CCQ; Significant differences (post hoc analysis) are indicated with a solid line.

The former GOLD classification shows a gradual but substantially overlapping decline in level of PA and accordingly a linear increase in proportion of inactive patients in the higher stages. In all multidimensional classifications patients in the highest quadrants (D) are less active and have a lower 6MWD compared to those in quadrant A (p<0.001). Using mMRC shows a lower PA and decreased 6WMD in quadrant D vs C (p = 0.04 and p<0.001 respectively) and B vs A (p = 0.02 and p<0.001 respectively), see Fig 2 and Table 2. The proportion of active patients was the highest (≈2/3 of patients) in GOLD I and GOLD A quadrants, irrespective of the classification. Of all patients classified as inactive (n = 80), 16% was included in GOLD stage IV whereas 49–54% (using mMRC, CAT or CCQ) are allocated in GOLD quadrant D. The sensitivity analyses using minimal PA restrictions (including weekend days) showed comparable results (see S1 File, S2 Fig).

Patients classified as former GOLD stage III-IV (n = 50) show lower PA levels compared to those in stages I-II (n = 86) (3698 [2865] vs. 5080 [4157] steps.day^-1^, p<0.001). Similarly, patients in quadrants C-D (n = 75) present with a lower step count compared to those quadrants A-B (n = 61) (3824 [2976] vs. 5508 [4672] steps.day^-1^, p<0.001).

**Fig 3:**
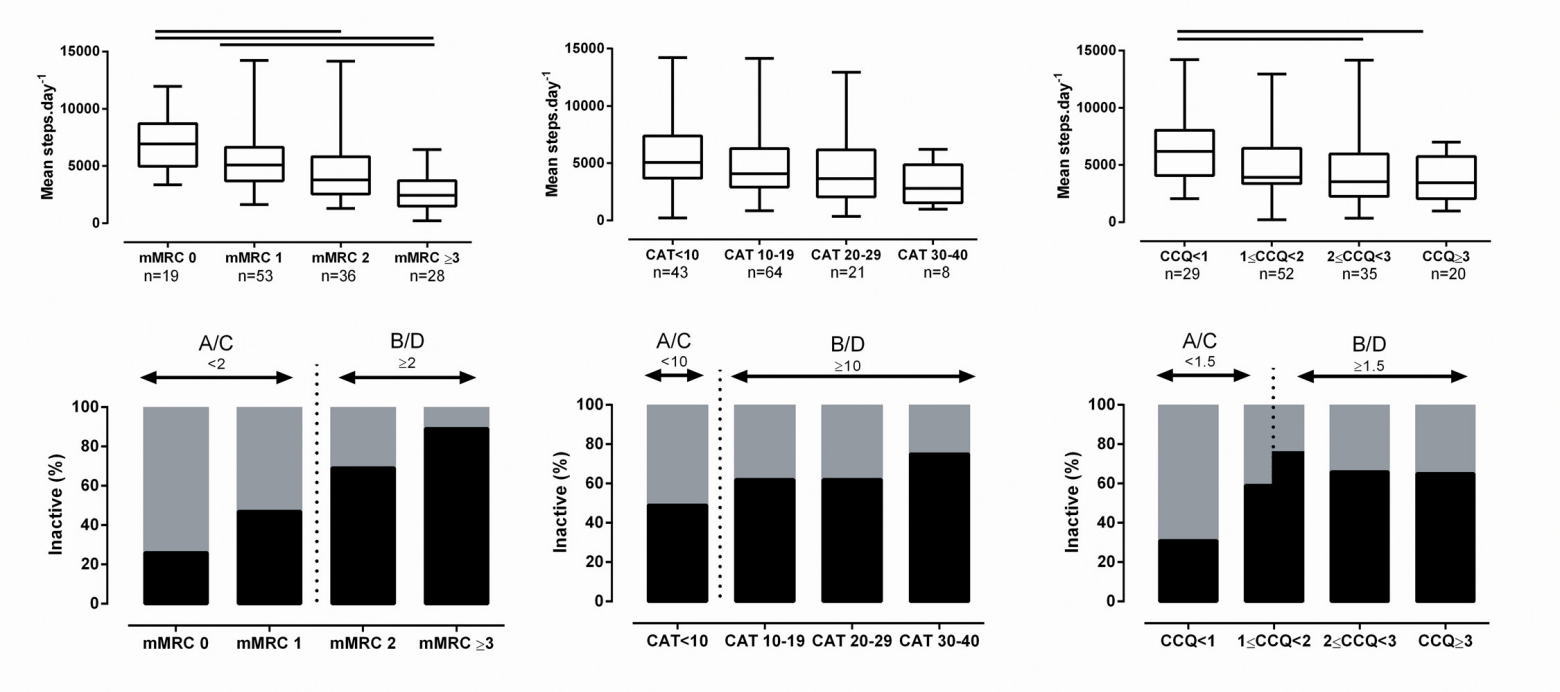
Physical activity across different mMRC, CAT and CCQ cut-offs. Upper panels present physical activity data, significant differences (post hoc analysis) are indicated with a solid line; lower panels depict proportion of patients defined as sedentary (black bars). Dotted line present cut off used in the GOLD classification; A/C and B/D indicate respectively quadrants A or C and B or D.

The impact of having symptoms on PA depends on the questionnaire used in the classification (less vs. more symptoms; mMRC: 3430 [2537] vs. 5443 [3776] steps.day^-1^,p<0.001; CAT 3835 [3616] vs.5070 [3681] steps.day^-1^, p = 0.02; CCQ 3714 [3240] vs. 5278 [4253] steps.day^-1^,p<0.001). PA was notably lower in patients with a higher mMRC score. Fig 3 depicts the impact of using mMRC, CAT and CCQ categorisation on the PA level.

### What is the agreement between the different classifications?

**Fig 4:**
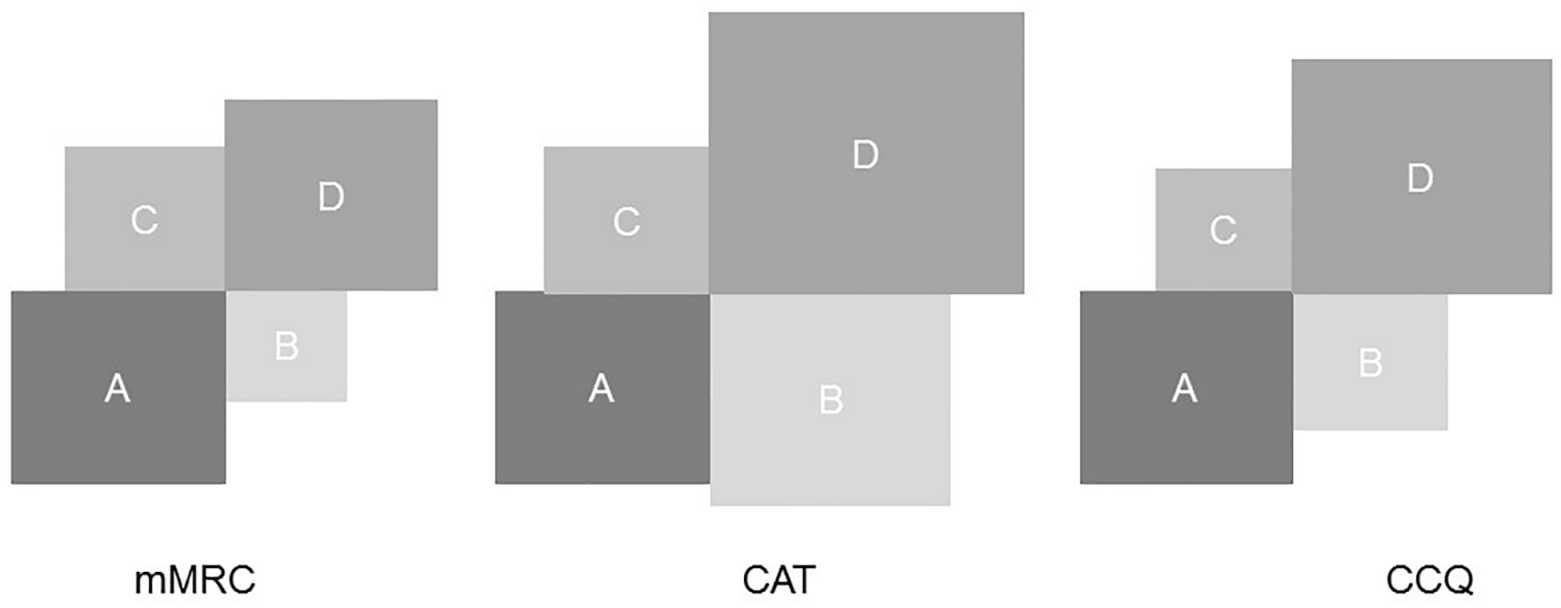
Patient distribution across the different GOLD classifications. The size of the squares represent the patient distribution relative to quadrant A. mMRC = combined assessment using mMRC; CAT = combined assessment using CAT; CCQ = combined assessment using CCQ.

Using mMRC, CAT or CCQ questionnaire to define symptoms resulted in different classifications of patients (Table 3 and Fig 4) The agreement between the classification of patients, based on mMRC and CAT questionnaire was moderate (κ = 0.57 [95% CI 0.47–0.67]). The agreement between the classification based on CCQ questionnaire and respectively the classification based on mMRC and CAT questionnaire was substantial (κ = 0.71 [95%CI 0.62–0.80] and κ = 0.72 [95%CI 0.63–0.80]).

**Table 3:** Frequency distribution of patients according to different GOLD classifications.

	**I (FEV**_**1**_**≥80%)**	**II(50%≤FEV**_**1**_**<80%)**	**III (30%≤FEV**_**1**_**<50%)**	**IV (FEV**_**1**_**≤30%)**
**GOLD**	19 (14%)	67 (49%)	35 (26%)	15 (11%)
	**A (Low R, Low S)**	**B (Low R, High S)**	**C (High R, low S)**	**D (High R, High S)**
**mMRC**	46 (34%)	15 (11%)	26 (19%)	49 (36%)
**CCQ**	40 (29%)	21 (15%)	16 (12%)	59 (43%)
**CAT**	27 (20%)	34 (25%)	16 (12%)	59 (43%)

GOLD = (former) spirometric GOLD classification, mMRC = combined assessment using mMRC questionnaire, CAT = combined assessment using CAT scores, CCQ = combined assessment using CCQ questionnaire; Low R = low severity on risk assessment; High R = high severity on risk assessment; Low S = less symptoms; High S = more symptoms

## Discussion

Our results show that patients identified by the multidimensional GOLD classification as being at high risk also have severely decreased PA level. However, the data also show that the use of different questionnaires results in a different patient distribution with different clinical characterization. Moreover, PA is notably lower in patients with a higher mMRC score, both in the low and high risk quadrants. Importantly, independent of the questionnaire used, approximately 30% of patients in quadrant A (i.e. low risk, low symptoms) show inactivity and 27 out of 80 (34%) inactive patients are classified in the low risk quadrants.

PA decreases with worse lung function [7,10]. In the multidimensional GOLD classification, by adding the history of exacerbations, more patients are shifted to the higher risk categories [5]. The very low PA seen in these upper quadrants, consistently related to the risk for an exacerbation and mortality [6], confirms the ability of the GOLD classification to identify severe patients, in need of maximal therapy. According to the GOLD guidelines, these patients require a maximal non-pharmacological treatment strategy including pulmonary rehabilitation and PA management [2].

COPD patients experience multiple symptoms, such as dyspnea, fatigue, anxiety and depression, influencing physical and social functioning [28]. Dyspnea, the major cause of disability in COPD, has already shown to be a better predictor of future mortality compared to severity of lung function [29]. Previous research showed a 5 to 8 fold increase in risk of cardiovascular and cancer mortality in quadrants B and D compared to A and C [30] and the highest prevalence of comorbidities and persistent inflammation was observed in patients in quadrant B [31], using mMRC in the classification. These data are in line with the present results and current literature showing an important link between physical (in)activity, important comorbidities [32] and related mortality [33]. Our data by showing a decreased physical activity in patients with higher mMRC score, independent of the risk assessment, supports the idea that mMRC is an important predictor of mortality [29], is mediated by a decreased PA in these patients.

An important aim of the GOLD strategy is to guide treatment of COPD patients. In patients at high risk, adding the symptom classification does not introduce significant changes to the (maximal) treatment decisions. However, in patients at low risk (e.g. patients in primary care) classifying patients in quadrant A or B will change the recommended treatment choice (e.g. starting rehabilitation) [2]. Our data suggest that the classification using mMRC would provide a better discrimination for the need for this non-pharmacological therapy. The different questionnaires will result in different patient distributions, leading to different clinical characteristics such as PA and 6MWD in the quadrants. Previous research showed the difference in patient distribution [12,13], but none investigated the differences in clinical characteristics, important for non-pharmacological treatment decisions. Although it could be argued that the multidimensional classification was proposed for disease management and not for prognostic purpose, interestingly, Casanova et al. showed the classification using mMRC was superior in the ability to predict mortality than the CAT and CCQ (using a cut-off of 1.0)[34]. These data could be related to our results showing the differences in PA across these classifications. Additionally, our data show the possible impact on non-pharmacological treatment decisions according to the choice of questionnaire. Taking all this into account, our data clearly echo the claim of Agusti, et al. that standardization of the choice of questionnaire to attribute the GOLD strata is needed [35].

Previous research reporting on PA across the different quadrants are mainly based on mMRC to classify patients and only measured PA at one time point. Moreira and colleagues did not find any difference in objectively measured activity time between quadrants B to D nor between the former stages [36], the latter is in contrast with available literature [10]. In line with our results, lower levels of PA of patients in quadrant B and D were concluded in the study of Boland,et al. [37], although the methodology of PA measurement was not specified. PA data across GOLD quadrants using the St. George’s Respiratory Questionnaire (SGRQ) as a surrogate for CAT showed the lowest PA level in patients in quadrant D. These authors reported a higher PA level in patients in quadrant C compared to B (although significance was not reported), in contrast with our results. This difference could, besides a difference in the questionnaire used (SGRQ), be explained by the low number of patients in quadrant C (5% of the sample) or the differences in PA monitoring properties and analysis [38].

A review by the GOLD scientific committee recommended studies comparing the specific symptoms-evaluating tools [35]. To the best of our knowledge this is the first study investigating the differences between the combined GOLD classifications, using each one of the three permitted questionnaires to evaluate symptom experience, in physical activity and exercise capacity characteristics of patients. Based on our data we hypothesize a need for different therapeutic strategies in terms of non-pharmacological treatment of patients with COPD in the different GOLD quadrants, depending on the classification, particularly in the low risk quadrants. Future research is needed to evaluate whether these treatment choices would yield different benefits.

Physical activity was objectively measured using an accelerometer validated for use in patients with COPD [19,20]; self-reported PA is known to relate poorly to objectively measured data [39]. With the aim of decreasing variability, a valid physical activity measurement was defined as having at least 2 weekdays of measurement on both time points [21]. Nevertheless because physical activity has shown to be lower during weekends compared to weekdays [40], we repeated the analyses including weekends (sensitivity analysis, see S1 File) to account for potential misclassification in relation to decisions about PA, which resulted in similar conclusions. We have chosen to use the number of steps as outcome measurement, known to be a more sensitive outcome in this patients population compared to e.g. time in moderate physical activity and lowered seasonal influence by combining 2 measurement periods [21]. Including patients in different European centers, representing a wide range of severity and physical activity, improves the representation of the COPD population. When compared to 3 large cohorts (ECLIPSE, Cocomics, COPDgene), using the mMRC in the classification [35], the present study shows a very similar distribution (A 31%, B 17%, C 16%, D 37%).

## Conclusions

The present study shows that patients identified by the multidimensional GOLD classification as being at high risk also have severely decreased physical activity levels. Using different questionnaires in the classification changes both the patient distribution and results in different clinical characteristics. We therefore believe that standardization of the questionnaire used for GOLD classification is critical first to allow fair comparison of different studies using this as an entry criterion and second to be used as base for treatment recommendations. Our data suggest that the mMRC may be the most useful. Lastly we highlight that a significant minority of patients in quadrant A have reduced PA emphasizing the importance of PA promotion even in milder and apparently asymptomatic patients.

## Supporting Information

S1 Fig Study flowchart. (JPG)

S2 Fig Physical activity across different GOLD classifications. (TIF)

S1 File Data supplement including power calculation, characteristics of the excluded patients, clinical features of patients across the spirometric GOLD stages and physical activity sensitivity analyses. (DOCX)
