# Supplementary figures and images for: Physical Activity Characteristics across GOLD Quadrants Depend on the Questionnaire Used
Source: PLoS One. 2016 Mar 14;11(3):e0151255. doi: 10.1371/journal.pone.0151255 (PMC4790973; doi:10.1371/journal.pone.0151255)

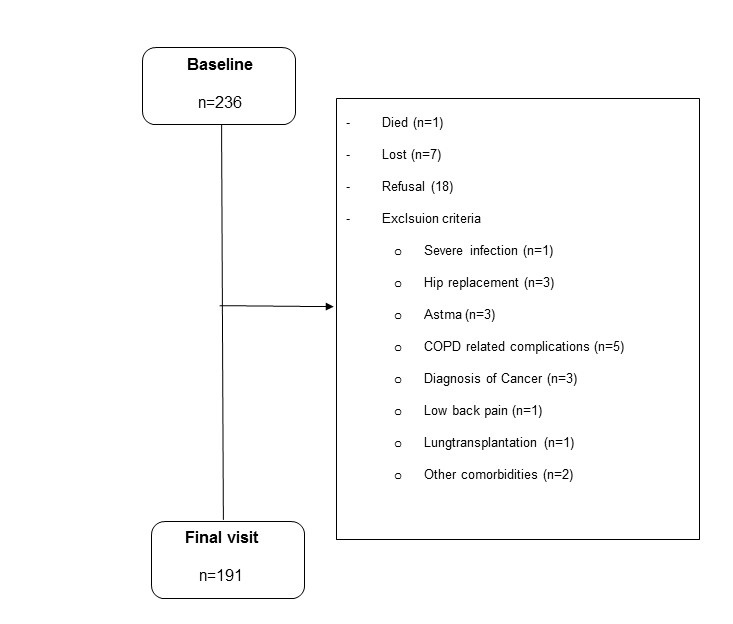

Supplement: S1 Fig — (JPG) [file pone.0151255.s001.jpg]

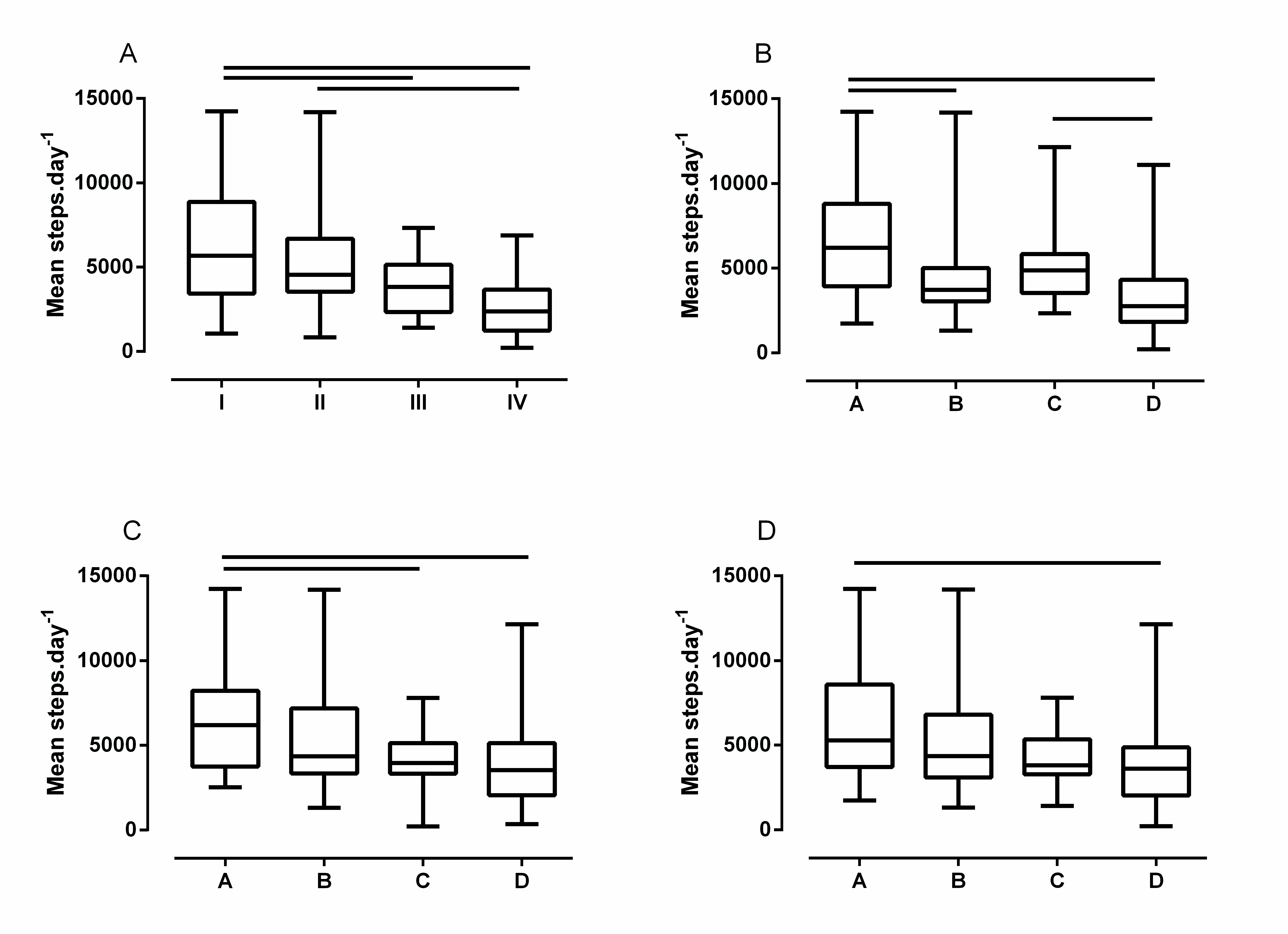

Supplement: S2 Fig — (TIF) [file pone.0151255.s002.tif]
