# Supplementary material for: Physical Activity Characteristics across GOLD Quadrants Depend on the Questionnaire Used
Source: PLoS One. 2016 Mar 14;11(3):e0151255. doi: 10.1371/journal.pone.0151255 (PMC4790973; doi:10.1371/journal.pone.0151255)
Supplement: S1 File — (DOCX) [file pone.0151255.s003.docx]

# Physical activity characteristics across GOLD quadrants depend on the questionnaire used

# Online supplement

## Power calculation

A power calculation (analysed with G*Power 3.1.6[1]) focused on the primary aim of this investigation - the discrimination in physical activity (PA) accross the four (A/B/C/D) new GOLD quadrants. The sample size calculation was based on data available on the discrimination of PA across the former spirometric classification (mean steps GOLD I/II/III/IV: 7990 / 7190 / 5126 / 2773 steps with a SD within groups of 3692[2] to achieve a power of 0.8 (F- test, One way ANOVA, p=0.05) and resulted in a total sample size of 44 patients.

## General patient characteristics

A total of 191 patients with COPD completed the whole trial and were considered for inclusion in the present study. A total of 236 patients were recruited one year before and 45 patients dropped out the study (S1 Fig.).

**S1 Fig. Study flowchart**

Of the 169 patients with all GOLD classifiers, 33 patients were excluded from the analysis because of no valid PA measurement. These patients did not differ from those included in the present analyses (Table A).

Table A. Patient characteristics of patients included and excluded in the analyses.

| **Variable** | **Included patients (n=136)** | **Excluded patients (n=33)** | **p-value** |
| --- | --- | --- | --- |
| Female/male* | 34 (25%) / 102 (75%) | 9 (27%) / 24 (73%) | 0.79 |
| Age (y) | 68 ± 8 | 69±8 | 0.34 |
| BMI (kg.m^-2^) | 27 ± 5 | 25±4 | 0.09 |
| FEV_1_ (%pred) | 58 ± 21 | 57±27 | 0.83 |
| 6MWD (m) | 444 ± 129 | 418±149 | 0.32 |
| CAT score | 14 ± 8 | 17±8 | 0.08 |
| CCQ score | 1.8 [± 1.0 | 2.0±1.1 | 0.40 |
| mMRC (0/1/2/3/4)* | 19/ 53/ 36 / 27 / 1 (14%/39%/26%/20%/1%) | 1/13/11/8/0 (3%/40%/33%/24%/0%) | 0.46 |
| Active smokers* | 18 (13%) | 7 (21%) | 0.25 |
| Exacerbations (n.y^-1^)^&^ | 1 (0-12) | 1 (0-10) | 0.84 |
| Hospitalizations due to exacerbation (n.y^-1^) ^&^ | 0 (0-4) | 0 (0-10) | 0.08 |

Data are presented as mean ± SD, *=data presented as n(%),^&^data presented as median (min-max) ; p-value results from an unpaired ttest or chi square test(*)

What are the patients’ characteristics according to the different classifications?

Table B. Patient characteristics across the spirometric GOLD stages

|  | **Spirometric GOLD classification** | | | | |
| --- | --- | --- | --- | --- | --- |
|  | I (n=19) | II (n=67) | III (n=35) | IV (n=15) | P-value |
| FEV_1_ (%pred) | 92±13 | 65±9^I^ | 41±5 ^I,II^ | 24±5^I,II,III^ | **<0.001** |
| mMRC (score) | 0.7±0.8 | 1.4±0.9^I^ | 1.9±0.8^I,II^ | 2.6±0.7^I,II,III^ | **<0.001** |
| CAT (score) | 10±6 | 12±7 | 16±9^I,II^ | 20±6 ^I,II^ | **<0.001** |
| CCQ (score) | 1.1±0.7 | 1.6±0.9 | 2.2±1.0 ^I,II^ | 2.6±0.8 ^I,II^ | **<0.001** |
| Exacerbations (n.y^-1^)^&^ | 0[1] | 1[2] | 1[3] | 4[5] ^I,II^ | **<0.001** |
| Hospitalizations (n.y^-1^)^&^ | 0[0] | 0[0] | 0[0] | 0[1] | 0.08 |
| 6MWD (m) | 532±139 | 463±108 | 419±116 ^I^ | 300±111 ^I,II,III^ | **<0.001** |
| 6MWD (%pred) | 85±18 | 74±16 | 67±16 ^I^ | 47±16 ^I,II,III^ | **<0.001** |
| Inactive patients (%)* | 6(32) | 36(54) | 25(71)^I^ | 13(87)^I,II^ | **0.003** |

Data are presented as mean ± SD, *=data presented as n(%),^&^data presented as median [IQR]; p-value results from a one-way ANOVA, chi square test(*) or Kruskal Wallis (^&^); Post hoc analyses, adjusted for multiple testing: ^I^different from I (II, III or IV) ; ^II^different from II (III or IV); ^III^different from III (IV)

## Results with minimal PA restriction

The minimal PA restriction has been defined as an overall minimum of 2 days (with at least 8 hours of wearing time) to obtain reliable measurements, based on previous research[3], resulting in a total of 163 patients. Including these patient resulted in a comparable distribution across the quadrants compared to the main analyses (% of patients in A/B/C/D according to spirometric GOLD 16%/46%/26%/12%; mMRC combined classification 32%/12%/19%/37%; CAT combined classification 19%/25%/12%/44%; CCQ combined classification 28%/16%/14%/42%).

The different quadrants and GOLD stages did not differ in terms of wearing time (880±110min). Patients wore the monitor for 11±3 days. The proportion of patients defined as being ‘inactive’ (mean step count <4850) did differ between the GOLD stages and GOLD quadrants (p<0.01). Similar trends were seen in these proportions across the different classifications, compared to the main analysis, although slightly higher proportions of inactivity could be observed, mainly in quadrant A (proportion of inactivity (%) in A/B/C/D according to spirometric GOLD 42%/59%/74%/89%; mMRC combined classification 42%/74%/56%/83%; CAT combined classification 40%/59%/75%/74%; CCQ combined classification 46%/60%/65%/77%). PA across the quadrants is presented in Figure S2.

S2 Fig. Physical activity across different GOLD classifications. A= Spirometric GOLD classification; B= combined assessment using mMRC; C= combined assessment using CAT; D= combined assessment using CCQ; Significant differences (post hoc analysis) are indicated with a solid line.

Similarly to the main analysis, patients in the higher stages of the spirometric classification show lower PA levels compared to those in milder stages (3493 [2734] vs. 4659 [3705] steps per day, p<0.001) and those in the upper quadrants of the combined classification have a lower PA level compared to patients in the lower quadrants (3701 [2971] vs. 4836 [4267] steps per day, p<0.001). The impact of having more symptoms is as well comparable to the main analysis (mMRC: 3328 [2444] vs. 5197 [3571] steps.day^-1^,p<0.001; CAT 3848 [3493] vs.4694 [3586] steps.day^-1^, p=0.008; CCQ 3768 [3224] vs. 4649 [3602] steps.day-1,p<0.001)

## References

1. Faul F, Erdfelder E, Lang AG, Buchner A. G*Power 3: a flexible statistical power analysis program for the social, behavioral, and biomedical sciences*.* Behav Res Methods 2007; 39: 175-191.

2. Watz H, Waschki B, Boehme C, Claussen M, Meyer T, Magnussen H. Extrapulmonary effects of chronic obstructive pulmonary disease on physical activity: a cross-sectional study*.* Am J Respir Crit Care Med 2008; 177: 743-751.

3. Pitta F, Troosters T, Spruit MA, Probst VS, Decramer M, Gosselink R. Characteristics of physical activities in daily life in chronic obstructive pulmonary disease*.* Am J Respir Crit Care Med 2005; 171: 972-977.
